# Metabolomic profiles associated with physical activity in White and African American adult men

**DOI:** 10.1371/journal.pone.0289077

**Published:** 2023-11-09

**Authors:** Yan Du, Yuan-Yuan Li, Byeong Yeob Choi, Roman Fernadez, Kuan-Jui Su, Kumar Sharma, Lu Qi, Zenong Yin, Qi Zhao, Hui Shen, Chuan Qiu, Lan-Juan Zhao, Zhe Luo, Li Wu, Qing Tian, Hong-Wen Deng

**Affiliations:** 1 School of Nursing, University of Texas Health Science Center at San Antonio, San Antonio, Texas, United States of America; 2 Department of Nutrition, Nutrition Research Institute, University of North Carolina at Chapel Hill School of Public Health, Kannapolis, North Carolina, United States of America; 3 Department of Population Health Sciences, University of Texas Health Science Center at San Antonio, San Antonio, Texas, United States of America; 4 Tulane Center for Biomedical Informatics and Genomics, School of Medicine, Tulane University; New Orleans, LA, United States of America; 5 Center for Precision Medicine, School of Medicine, University of Texas Health Science Center at San Antonio, San Antonio, Texas, United States of America; 6 Department of Epidemiology, School of Public Health and Tropical Medicine, Tulane University; New Orleans, LA, United States of America; 7 Department of Public Health, University of Texas at San Antonio, San Antonio, TX, United States of America; 8 Department of Preventive Medicine, University of Tennessee Health Science Center, Memphis, TN, United States of America; Northeastern University, UNITED STATES

## Abstract

**Background:**

Physical activity (PA) is associated with various health benefits, especially in improving chronic health conditions. However, the metabolic changes in host metabolism in response to PA remain unclear, especially in racially/ethnically diverse populations.

**Objective:**

This study is to assess the metabolic profiles associated with the frequency of PA in White and African American (AA) men.

**Methods:**

Using the untargeted metabolomics data collected from 698 White and AA participants (mean age: 38.0±8.0, age range: 20–50) from the Louisiana Osteoporosis Study (LOS), we conducted linear regression models to examine metabolites that are associated with PA levels (assessed by self-reported regular exercise frequency levels: 0, 1–2, and ≥3 times per week) in White and AA men, respectively, as well as in the pooled sample. Covariates considered for statistical adjustments included race (only for the pooled sample), age, BMI, waist circumstance, smoking status, and alcohol drinking.

**Results:**

Of the 1133 untargeted compounds, we identified 7 metabolites associated with PA levels in the pooled sample after covariate adjustment with a false discovery rate of 0.15. Specifically, compared to participants who did not exercise, those who exercised at a frequency ≥3 times/week showed higher abundances in uracil, orotate, 1-(1-enyl-palmitoyl)-2-oleoyl-GPE (P-16:0/18:1) (GPE), threonate, and glycerate, but lower abundances in salicyluric glucuronide and adenine in the pooled sample. However, in Whites, salicyluric glucuronide and orotate were not significant. Adenine, GPE, and threonate were not significant in AAs. In addition, the seven metabolites were not significantly different between participants who exercised ≥3 times/week and 1–2 times/week, nor significantly different between participants with 1–2 times/week and 0/week in the pooled sample and respective White and AA groups.

**Conclusions:**

Metabolite responses to PA are dose sensitive and may differ between White and AA populations. The identified metabolites may help advance our knowledge of guiding precision PA interventions. Studies with rigorous study designs are warranted to elucidate the relationship between PA and metabolites.

## Introduction

Many studies have documented the benefits of physical activity (PA) in preventing and managing various chronic health conditions and reducing mortality [1]. These conditions include but are not limited to cardiovascular diseases [2], type 2 diabetes [1], metabolomic syndrome (particularly obesity and insulin resistance) [3], cognitive function [4], musculoskeletal health [5], and mental health [6]. However, the underlying mechanisms of the health benefits of PA are largely unknown. Emerging studies have examined the pathophysiological pathways of PA on health outcomes through metabolomics approaches [7].

Metabolomics is a novel approach to systematically profile metabolites in biofluids, cells, and tissues at the molecular level [8]. Metabolites represent the downstream expression of the genome, transcriptome, and proteome and the final endpoints of the interaction of the genes, environment and lifestyles [9]. Studying metabolomic signaling is one of the most powerful approaches to revealing inherent omics variation closest to the disease risk/phenotype [8]; it is promising and useful to elucidate the mechanisms that link lifestyle behaviors and guide precision lifestyle intervention, including PA [10–12].

Most previous studies examining PA and metabolomics assessed the acute influence of exercise on metabolites. After reviewing 27 studies, Schranner and colleagues reported that the abundance of 196 metabolites changed significantly within 24 hours after endurance or resistance exercise in at least two experiments [13]. Similarly, another review study concluded that most studies focused on metabolite changes after the bout (s) of exercise, and cross-sectional studies are also needed to understand the human system’s response to chronic PA workloads [14]. In addition, available studies that examined the effect of chronic PA on metabolites used samples of adults from all age groups, such as those aged 40 and older with a majority being 65 and older [15], and those aged 25 and over with mean age of around 55 years old [16]. Various factors may complicate assessing the associations of PA and metabolites in a population using a wide age range, such as the health conditions cumulated throughout the aging process. Furthermore, increasing numbers of studies reported the possibility of molecular differences (e.g., metabolites) between women and men, and gender differences might need consideration when developing personalized and precision treatment and intervention [17–20]. Assessing metabolites responses to PA in different genders is key to more precisely elucidating the relationship between metabolites and PA. Last but not least, African Americans (AAs) are disproportionally affected by various chronic diseases compared to other race/ethnicity groups [21]. For instance, previous studies reported that metabolites associated with type 2 diabetes [22,23], and the risk of coronary heart disease [23] differ between different racial/ethnical groups. The metabolomic differences may reflect the underlying variations of genetic, environmental, and behavioral factors (e.g., physical activity) [22,24,25]. However, PA-related metabolomic profiles in AAs are rarely studied [26]. Therefore, this study aimed to explore metabolites associated with PA in White and AA men aged between 20–50 years old.

## Materials and methods

### Study population

The current study included 698 White and African American participants with available metabolomic data from the ongoing Louisiana Osteoporosis Study (LOS) (from 2011 to current). LOS is a cross-sectional study that aims to build a large sample pool and database to investigate the genetic and environmental factors for complex chronic health conditions (e.g., osteoporosis, obesity, and sarcopenia) in Southern Louisiana. The inclusion and exclusion criteria of LOS have been previously described [27,28]. Each participant signed a consent form before any data collection. All participants included in this study were men between 20 and 50 years old. The Tulane University Institutional Review Board approved this study, and the principles of the Declaration of Helsinki were followed [29].

### Measurements

#### Physical activity

We measured participation in leisure-time PA with the self-reported number of times the participants exercised weekly. Several scientific articles used these PA measures [30,31]. According to the Physical Activity Guidelines for Americans 2nd edition [32], participating in leisure-time PA at least 3 days per week is associated with significant health benefits, and participating in any amount of PA (e.g., 1 to 2 times a week) yields more health benefits compared to no PA [33,34]. Therefore, in the current study, we classified levels of PA as 0, 1–2, and ≥3 times per week. PA is any body movement that increases energy expenditure; exercise is a subcategory of PA referred to as planned, structured, and repetitive body movements that focus on improving or maintaining physical fitness [35]. In this current study, we only assessed PA by self-reported exercise frequency per week. To be noted, both PA and exercise will be used throughout as this study assessed PA levels by self-reported exercise.

#### Demographics and other covariates

A questionnaire survey was used to collect age, gender, race/ethnicity, and other lifestyle behaviors. Self-reported race/ethnicity was identified by choosing from the following options: African American/Black, Asian, Caucasian/White, Hispanic/Latino, Native American/Pacific Islander, and others. Questions with yes/no responses assessed other behavioral factors regarding participant smoking status and alcohol consumption. Weight was measured in light indoor clothing using a calibrated balance beam scale, and height was measured using a calibrated stadiometer without shoes. We calculated body mass index (BMI) as weight (kg) divided by height squared (m^2^).

#### Metabolomic analyses

We collected blood sample from each participant, centrifuged the blood sample at 1000G for 5 minutes, aliquoted the serum into 2-mLmicrotubes, and stored the serum at -80°C before further processing. Metabolon (Durham, NC) analyzed the serum samples of the current study. Samples were prepared using the automated MicroLab STAR® system from Hamilton Company. Specifically, to remove protein, dissociate small molecules bound to protein or trapped in the precipitated protein matrix, and recover chemically diverse metabolites, proteins were precipitated with methanol under vigorous shaking for 2 min (Glen Mills GenoGrinder 2000) followed by centrifugation. The resulting extract was divided into five fractions for different types of analyses and backups. Samples were placed briefly on a TurboVap® (Zymark) to remove the organic solvent. The sample extracts were stored overnight under nitrogen before preparation for analysis.

Several types of controls were analyzed in concert with the experimental samples, which included: a pooled matrix sample generated by taking a small volume of each experimental sample to serve as a technical replicate throughout the data set; extracted water samples to serve as process blanks; and a cocktail of qualitative control (QC) standards carefully chosen to not interfere with the measurement of endogenous compounds were spiked into every analyzed sample, and allowed for instrument performance monitoring and aided chromatographic alignment.

All methods utilized a Waters ACQUITY ultra-performance liquid chromatography (UPLC) and a Thermo Scientific Q-Exactive high resolution/accurate mass spectrometer interfaced with a heated electrospray ionization (HESI-II) source and Orbitrap mass analyzer operated at 35,000 mass resolution. All the samples were analyzed in both positive and negative ion modes with heated electrospray ionization. The mass spectroscopy (MS) analysis alternated between MS and data-dependent MS^n^ scans using dynamic exclusion. The scan range varied slightly between methods but covered 70–1000 m/z.

#### Bioinformatics

The informatics system consisted of 4 major components, the Laboratory Information Management System (LIMS), the data extraction and peak-identification software, data processing tools for QC and compound identification, and a collection of information interpretation and visualization tools for use by data analysts. The informatics component hardware and software foundations were the LAN backbone, and a database server running Oracle 10.2.0.1 Enterprise Edition.

Metabolon’s hardware and software extracted, peak-identified, and QC processed the raw data. Three criteria formed the basis of the biochemical identifications: retention index within a narrow retention time/index window of the proposed identification, accurate mass match to the library +/- 10 ppm, and the MS/MS forward and reverse scores between the experimental data and authentic standards. A comparison of the ions presenting in the experimental spectrum to the ions presenting in the library spectrum determined the MS/MS scores. While there may be similarities between these molecules based on one of these factors, using all 3 data points can distinguish and differentiate biochemicals.

The design of the QC and curation processes ensured accurate and consistent identification of true chemical entities and removal of those representing system artifacts, misassignments, and background noise. Peaks were quantified using area-under-the-curve. Data normalization step was performed to correct variation resulting from instrument inter-day tuning differences. Essentially, each compound was corrected in run-day blocks by registering the medians to equal one (1.00) and normalizing each data point proportionately. We identified a total of 1,378 biochemicals, with 1,133 compounds of known identities (named biochemicals) and 245 compounds of unknown structural identities (unnamed biochemicals).

### Statistical analysis

Kruskal-Wallis rank sum test was used to describe continuous sample characteristic variables; Pearson’s Chi-squared test was used to assess categorical sample characteristic variables. To identify metabolites significantly associated with PA, linear regression models were fitted using individual metabolites and PA levels as response and exposure variables, respectively,. We increased the number of covariates included in the regression models for statistical adjustments. Two dummy variables were used to model PA; each dummy variable compared PA performed 1–2 or ≥3 times a week with 0 times per week. Metabolite abundances were log-transformed for regression analysis to reduce the impact of extreme values. To assess the association between each metabolite and PA levels, we fitted three regression models for each metabolite: Model 1 included PA levels only; Model 2 adjusted for race and age, which were non-modifiable factors; and Model 3 additionally adjusted for modifiable factors, including BMI, waist circumference, smoking, and alcohol use. We tested whether the exercise dummy variables’ regression coefficients were equal to 0, which implies no association between PA levels and individual metabolites. Based on Benjamini and Hochberg’s method to correct for multiple testing [36], we converted raw *p*-values of Wald statistics were converted to false discovery rates (FDR or q-values), and the q-values/FDR less than 0.15 were considered significant. All model fittings had no multicollinearity issues based on the variance inflation factors. For the 7 metabolites selected by Model 3, we conducted stratified analysis by race (African American and White) to investigate whether the associations between PA and those metabolites could change by different race groups. More specifically, we fitted Model 3 (without the race/ethnicity variable) to African Americans and Whites separately to examine race-specific associations.

### Pathway analysis

To identify pathways represented by the 7 significant metabolites, we conducted a pathway analysis using the web tool MetaboAnalyst 5.0 [37]. The pathway analysis integrated well-established (e.g., over-representation analysis) methods and novel algorithms and concepts (e.g., network topology analysis). The topology analysis accounts for the position of a metabolite in the pathway structure and provides an impact value ranging from 0 (minimum impact) to 1 (maximum impact) for each tested metabolite.

## Results

Table 1 shows the characteristics of the study population. The mean age of the sample was 36.9±8.3, and over 44.5% (297) were African American. Approximately 61.4% of the sample reported exercising ≥3 times/week. Those who reported 0 regular exercise engagement were relatively older (mean age = 38.1±8.2) compared to those who exercised 1–2 times/week (mean age = 36.4 ±8.0) and ≥3 times/week (mean age = 36.0 ±8.2). A higher proportion of Whites (64%) reported exercising ≥3 times/week compared to African Americans (36%). The mean BMI (28.0 ±5.8) and waist circumference (86.0±14.0) were the lowest in those who exercised ≥3 times/week. Over 67% of those who exercised ≥3 times/week reported alcohol consumption compared to 54% of those who reported no regular exercise. We further assessed the sample characteristics by race/ethnicity groups and physical activity levels (Table 2). There was a trend that those who reported exercising ≥3 times/week were younger (mean age = 38.0 ±8.0), had the lowest BMI (27.0 ±5.2), and waist circumference (85.4±14.0), but the differences were not significant in AAs (p>0.05); On the other hand, it is all significant in Whites with p = 0.005 for age difference, p = 0.002 for BMI, and p<0.001 for waist circumference.

**Table 1:** Characteristics of the sample by race/ethnicity groups.

Characteristic	Overall	0 times/week N = 185 (28.8%)	1–2 times/week N = 72 (10.8%)	≥3 times/week N = 410 (61.4%)	P-value
Age (years)	36.9 (8.3)	38.3 (8.1)	36.4 (8.2)	36.4 (8.3)	0.02
Age group					0.14
Middle	47 (25%)	18 (25%)	137 (33%)	47 (25%)	
Young	138 (75%)	54 (75%)	273 (67%)	138 (75%)	
Race					<0.001
African American	297 (45%)	100 (54%)	31 (43%)	146 (36%)	
White	370 (56%)	85 (46%)	41 (57%)	264 (64%)	
BMI	27.2 (5.3)	28.0 (5.8)	27.9 (5.1)	26.8 (5.0)	0.02
BMI group					0.09
Normal	50 (27%)	13 (18%)	84 (20%)	50 (27%)	
Overweight/Obese	135 (73%)	59 (82%)	326 (80%)	135 (73%)	
Waist circumference (cm)	87.0 (14.9)	89.0 (14.9)	89.8 (18.6)	85.8 (14.0)	0.021
Smoking status					0.7
No	314 (45%)	79 (43%)	35 (49%)	185 (45%)	
Yes	384 (55%)	106 (57%)	37 (51%)	225 (55%)	
Alcohol drinking					0.007
No	250 (37%)	83 (46%)	26 (36%)	131 (33%)	
Yes	434 (63%)	97 (54%)	46 (64%)	271 (67%)	

Mean (SD); n (%).

BMI = Body mass index.

Age group: Young included those age between 18–44 years old; Middle included those ≥45. BMI group included Normal with BMI<25 and Overweight/Obese with BMI ≥25.

Kruskal-Wallis rank sum test was used for continuous independent variables; Pearson’s Chi-squared test was used for categorical independent variables.

**Table 2:** Characteristics of the sample by race/ethnicity groups and physical activity levels.

Characteristic	African American				White			
	0 time/week	1–2 times/week	≥3 times/week	P-value	0 times/week	1–2 times/week	≥3 times/week	P-value
Age (years)	38.0 (7.9)	39.0 (7.9)	38.0 (7.5)	0.7	38.6 (8.4)	34.4 (7.9)	35.5 (8.5)	0.005
Age group				0.8				0.039
Mid	26 (26%)	9 (29%)	34 (23%)		24 (28%)	4 (9.8%)	50 (19%)	
Young	74 (74%)	22 (71%)	112 (77%)		61 (72%)	37 (90%)	214 (81%)	
BMI	27.3 (5.6)	27.4 (4.8)	27.0 (5.2)	0.8	28.9 (5.9)	28.4 (5.3)	26.7 (4.8)	0.002
BMI group				0.4				0.05
Normal	31 (31%)	7 (23%)	51 (35%)		16 (19%)	11 (27%)	86 (33%)	
Overweight/Obese	69 (69%)	24 (77%)	95 (65%)		69 (81%)	30 (73%)	178 (67%)	
Waist circumference (cm)	85.8 (14.5)	90.3 (23.0)	84.7 (14.2)	0.5	92.8 (14.6)	89.4 (14.7)	86.4 (13.9)	<0.001
Smoking status				0.5				0.2
No	45 (45%)	13 (42%)	55 (38%)		34 (40%)	22 (54%)	130 (49%)	
Yes	55 (55%)	18 (58%)	91 (62%)		51 (60%)	19 (46%)	134 (51%)	
Alcohol drinking				0.057				0.6
No	57 (59%)	13 (42%)	63 (44%)		26 (31%)	13 (32%)	68 (26%)	
Yes	40 (41%)	18 (58%)	80 (56%)		57 (69%)	28 (68%)	191 (74%)	

Mean (SD); n (%).

BMI = body mass index.

Age group: Young included those age between 18–44 years old; Middle included those ≥45. BMI group included Normal with BMI<25 and Overweight/Obese with BMI ≥25.

Kruskal-Wallis rank sum test was used for continuous independent variables; Pearson’s Chi-squared test was used for categorical independent variables.

**Fig 1:**
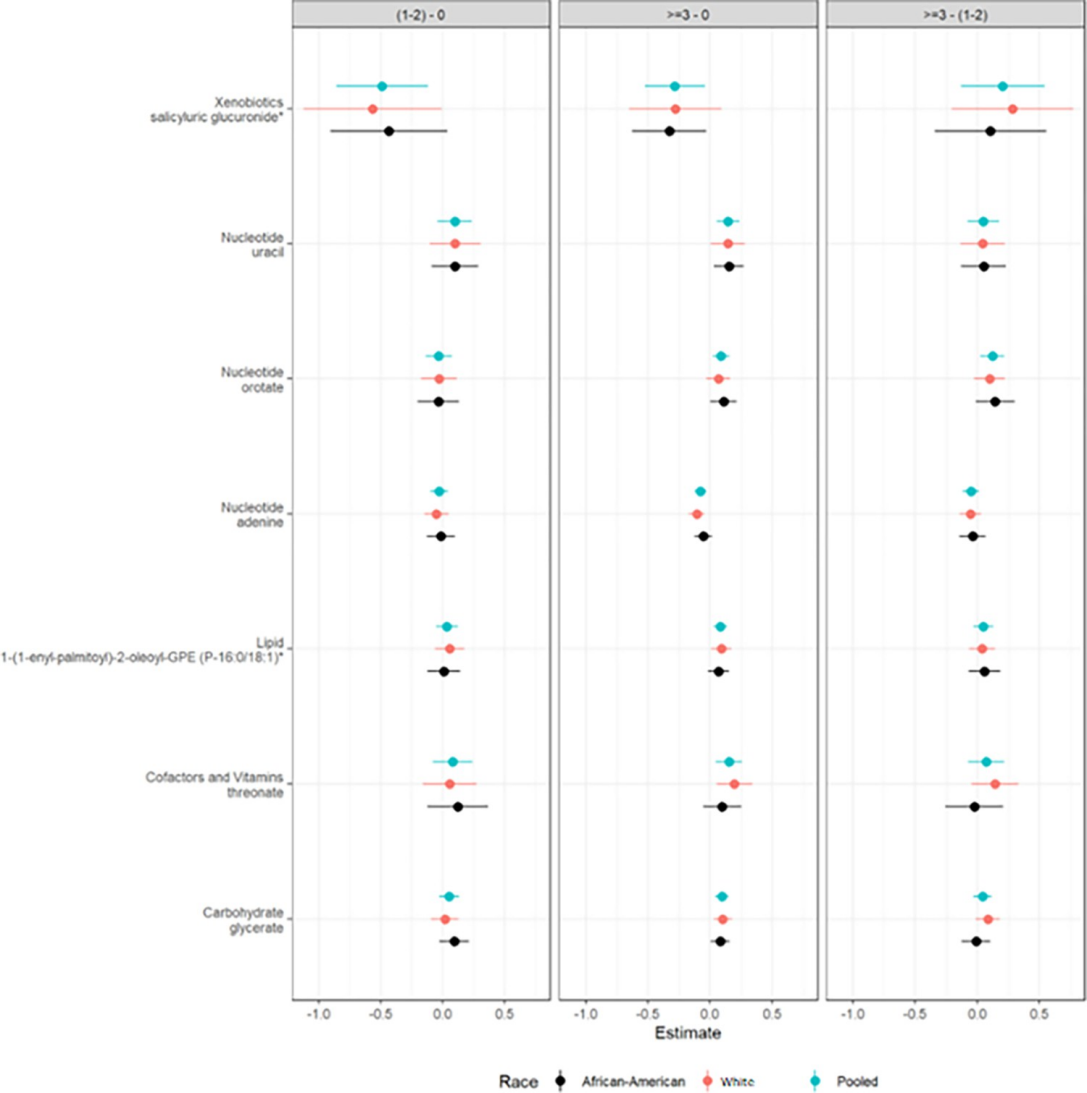
Seven metabolites associated with regular exercise adjusted for age, body mass index, waist circumference (cm), smoking, and alcohol; plus race for the pooled estimate. From left to right, pairwise groups comparisons are displayed for 1–2 versus 0 (reference), ≥**3** versus 0 (reference), and ≥**3** versus 1–2 (reference). Point estimates and 95% confidence intervals are presented for the mean differences in individual metabolite concentrations between the two exercise groups. The value 0 indicates no mean difference between the groups.

A total of 365 metabolites were related to PA levels (Table 3) before adjusting for any covariates. Of the 365 metabolites, we identified 105 metabolites with significant associations with PA levels (Table 3) after controlling for non-modifiable factors (race and age). After adjusting for both non-modifiable factors (race and age) and modifiable factors (BMI, waist circumference, smoking status, and alcohol drinking), 7 metabolites remained significant (salicyluric glucuronide, uracil, orotate, adenine, GPE, threonate, and glycerate) (Fig 1). The 7 metabolites associated with PA included 1 carbohydrate, 1 cofactor and vitamin, 3 nucleotides, 1 xenobiotic, and 1 lipid (Table 3).

**Table 3:** Distributions of metabolites detected by chemical class, and metabolites that were significantly associated with physical activity levels.

Chemical class					Metabolites associated with PA levels (after adjustment)		
	Total number of metabolites	Metabolites associated with PA levels (Model 1)		(Model 2)		(Model 3)	
Energy	11	5		1		0	
Partially Characterized Molecules	23	7		2		0	
Carbohydrate	27	6		1		1	
Cofactors and Vitamins	35	12		7		1	
Nucleotide	38	14		4		3	
Peptide	46	15		2		0	
Amino Acid	226	72		24		0	
Xenobiotics	252	82		19		1	
Lipid	452	152		45		1	
Total	1133	365		105		7	

BMI = body mass index.

Linear regression models were fitted using individual metabolites and PA levels as response and exposure variables, respectively. Model 1 included PA levels only, Model 2 adjusted for race and age, which were non-modifiable factors, and Model 3 additionally adjusted for modifiable factors, including body mass index, waist circumference, smoking, and alcohol use.

**Fig 2:**
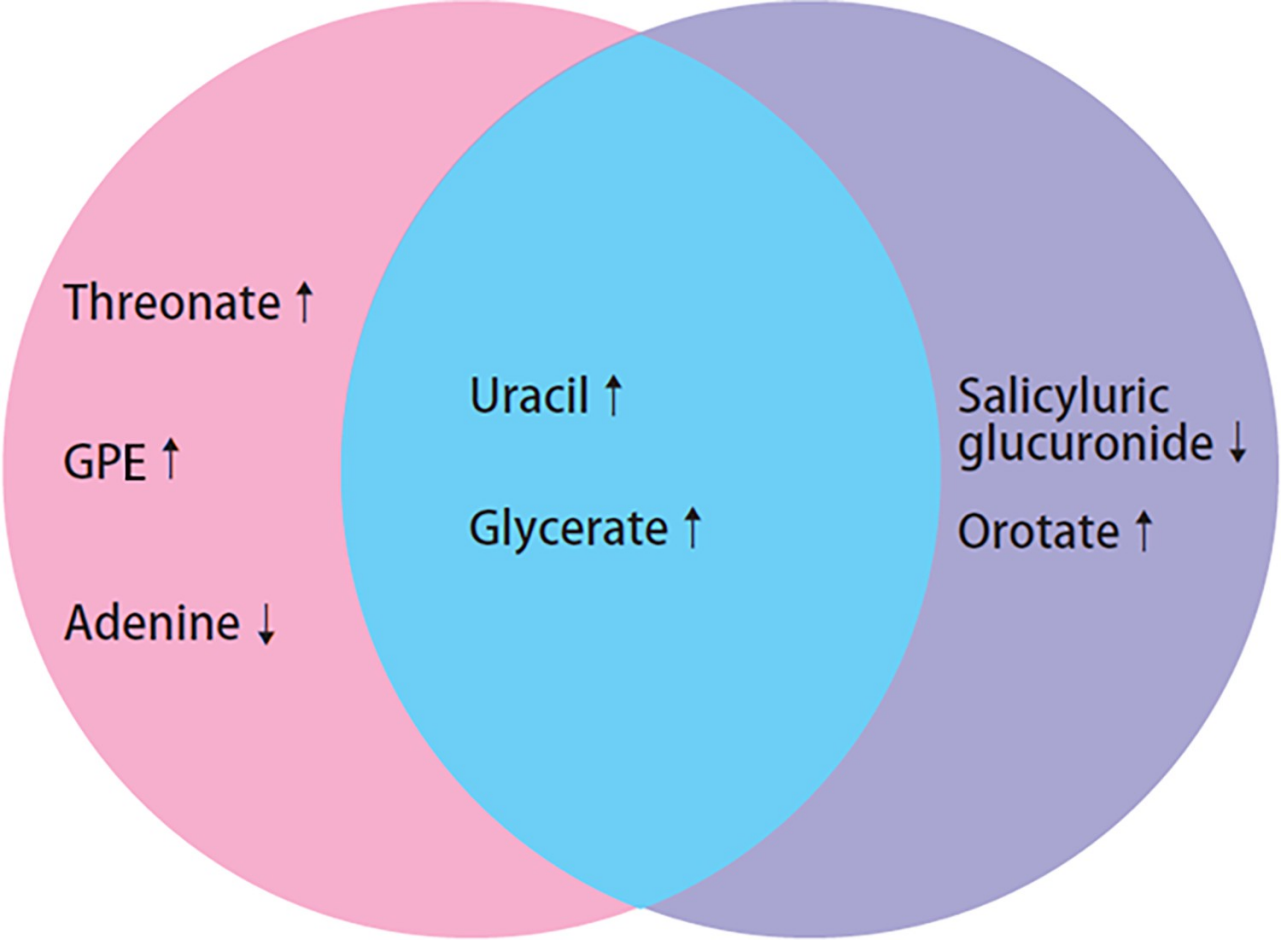
Veen diagram of significant alternated metabolites between those exercised ≥3 times/week and those reported no regular exercise in Whites (pink color) and AAs (purple), respectively, and in both Whites & AAs (blue). Up arrow indicates higher exercise frequency with higher metabolite concentration, and lower arrow indicates higher exercise frequency with lower metabolite concentration. GPE = 1-(1-enyl-palmitoyl)-2-oleoyl-GPE (P-16:0/18:1).

Interestingly, the abundances of all 7 metabolites differed between those who reported no exercise and those who exercised ≥3 times/week in the pooled sample (Table 4 and Fig 1). Specifically, those who exercised ≥3 times/week had higher uracil, orotate, GPE, threonate, and glycerate compared to those who reported no exercise. Those who exercised ≥3 times/week had lower abundances of salicyluric glucuronide and adenine than those who reported no exercise. For the respective White and AA groups who reported exercising ≥3 times/week and those who reported no regular exercise, salicyluric glucuronide and orotate were significant only in AAs; adenine, 1-(1-enyl-palmitoyl)-2-oleoyl-GPE (P-16:0/18:1) (GPE) and threonate were significant in Whites; uracil and glycerate were significant in both AAs and Whites (Fig 2). Although the trends of metabolite alterations with PA levels were not significant in some comparison groups in Whites or AAs, the trends moved in the same direction among all examined comparison groups (Fig 2).

**Table 4:** Seven metabolites associated with physical activity levels.

Metabolites	Sample	1-2/week VS. 0/week Coefficients (95% CI)	≥3/week VS. 0/week Coefficients (95% CI)	≥3/week VS. 1-2/week Coefficients (95% CI)
Xenobiotics Salicyluric glucuronide	Pooled	-0.489 (-0.857, -0.120)	-0.283 (-0.524, -0.041) *	0.206 (-0.132, 0.544)
	AA	-0.434 (-0.904, 0.035)	-0.328 (-0.627, -0.030) *	0.106 (-0.344, 0.556)
	White	-0.566 (-0.122, -0.009)	-0.281 (-0.653, 0.091)	0.285 (-0.203, 0.772)
Nucleotide Uracil	Pooled	0.097 (-0.043, 0.237)	0.146 (0.054, 0.237) **	0.048 (-0.080, 0.177)
	AA	0.100 (-0.089, 0.289)	0.152 (0.032, 0.272) **	0.052 (-0.129, 0.234)
	White	0.100 (-0.105, 0.305)	0.144 (0.007, 0.281) *	0.044 (-0.136, 0.223)
Nucleotide Orotate	Pooled	-0.032 (-0.138, 0.074)	0.090 (0.020, 0.160) *	0.122 (0.025, 0.219) *
	AA	-0.035 (-0.201, 0.131)	0.110 (0.005, 0.216) *	0.145 (-0.014, 0.304)
	White	-0.301 (-0.173, 0.112)	0.068 (-0.028, 0.163)	0.098 (-0.027, 0.223)
Nucleotide Adenine	Pooled	-0.028 (-0.100, 0.043)	-0.078 (-0.125, -0.031) ***	-0.050 (-0.116, 0.016)
	AA	-0.015 (-0.127, 0.097)	-0.053 (-0.124, 0.019)	-0.038 (-0.145, 0.070)
	White	-0.052 (-0.148, 0.045)	-0.107 (-0.171, -0.043) ***	-0.056 (-0.140, 0.029)
Lipid 1-(1-enyl-palmitoyl)-2-oleoyl-GPE (P-16:0/18:1)	Pooled	0.034 (-0.053, 0.121)	0.082 (0.025, 0.139) **	0.048 (-0.032, 0.127)
	AA	0.010 (-0.123, 0.143)	0.068 (-0.016, 0.153)	0.058 (-0.069, 0.186)
	White	0.055 (-0.063, 0.172)	0.093 (0.015, 0.172) *	0.039 (-0.065, 0.142)
Cofactors and Vitamins Threonate	Pooled	0.079 (-0.080, 0.238)	0.152 (0.048, 0.256) **	0.073 (-0.073, 0.218)
	AA	0.122 (-0.121, 0.365)	0.099 (-0.056, 0.253)	-0.023 (-0.256, 0.210)
	White	0.057 (-0.158, 0.272)	0.198 (0.054, 0.341) **	0.141 (-0.048, 0.329)
Carbohydrate Glycerate	Pooled	0.052 (-0.028, 0.132) **	0.095 (0.043, 0.148) ***	0.043 (-0.030, 0.117)
	AA	0.092 (-0.028, 0.213)	0.083 (0.006, 0.159) *	0.009(-0.125, 0.106)
	White	0.018 (-0.092, 0.127) *	0.103 (-0.030, 0.0.177) **	0.085 (-0.011, 0.182)

AA = African American.

Linear regression models were used with individual metabolites and PA levels as response and exposure variables, respectively. Adjustments were made for age, body mass index, waist circumference, smoking, and alcohol use, plus race/ethnicity for the pooled sample. Unstandardized coefficients were displayed for 1–2 versus 0 (reference), ≥3 versus 0 (reference), and ≥3 versus 1–2 (reference).

*p<0.05

**p<0.01

***<0.001

We found that for either pooled outcomes or separated outcomes for Whites and AAs (Table 4 and Fig 1), there were no significant differences in the abundances of the 7 metabolites between those who reported no exercise and those who exercised 1–2 times/week, as well as no differences between those exercised 1–2 times/week and exercised ≥3 times/week. In the pooled sample, however, we found significant differences in the salicyluric glucuronide between those who reported no exercise and those exercised 1–2 times/week and in the orotate between those exercised 1–2 times/week and ≥3 times/week in the pooled sample.

In the pathway analyses, 4 of the 7 PA-related metabolites (orotate, uracil, glycerate, and adenine) were annotated in the 8 metabolic pathways, and only 2 metabolic pathways (pyrimidine and glycerolipid metabolism) were significant with p<0.05 (Table 5). Of the 8 pathways, 3 included uracil, and 4 included glycerate. The impact values of the metabolites on these pathways varied (from almost 0 to 0.12).

**Table 5:** Pathway analysis results of alternated metabolites.

Pathways	Matched Metabolites		*P value* ^*a*^	*FDR*	Impact value ^b^
Pyrimidine metabolism		Orotate; Uracil	5.88E-03	4.94E-01	0.12
Glycerolipid metabolism		Glycerate	5.06E-02	1.00E+00	0.09
Pantothenate and CoA biosynthesis		Uracil	5.99E-02	1.00E+00	0.00
beta-Alanine metabolism		Uracil	6.60E-02	1.00E+00	0.00
Pentose phosphate pathway		Glycerate	6.91E-02	1.00E+00	0.00
Glyoxylate and dicarboxylate metabolism		Glycerate	9.92E-02	1.00E+00	0.08
Glycine, serine and threonine metabolism		Glycerate	1.02E-01	1.00E+00	0.02
Purine metabolism		Adenine	1.93E-01	1.00E+00	0.01

a. *P* values from the pathway enrichment analysis.

b. Impact values from the pathway topology analysis.

## Discussion

In the pooled sample, compared to those who did not exercise regularly, those who exercised ≥3 times/week had significantly higher abundances of uracil, orotate, GPE, threonate, and glycerate and significantly lower abundances of salicyluric glucuronide and adenine; however, the differences were not significant between the other PA comparison groups (0 vs. 1–2 times/week and 1–2 vs. ≥3 times/week). In addition, uracil and glycerate differed in both White and AA groups, but the other 5 metabolites were significantly different in either Whites or AAs. The findings suggest that PA-related metabolite changes might be dose sensitive and vary among racial/ethnic groups. Longitudinal studies in diverse race/ethnicity populations are needed to confirm the findings. In addition, race/ethnicity is not only a biological trait, but also a sociological trait [38,39]; a big data approach to comprehensively, dynamically, and relatively precisely collect all relevant data could be helpful in further advancing our knowledge and understanding of the human metabolomic profile and its responses to modifiable factors, such as lifestyle behaviors.

In the pooled and the respective racial/ethnical samples, those who exercised ≥3 times/week had significantly higher uracil and glycerate than those who did not exercise regularly. Uracil is in the pyrimidine metabolism pathway and belongs to the class of organic compounds known as pyrimidines. Uracil participates in several enzymatic reactions and evidence indicates a relationship to various health conditions. For example, a narrative review reported that many studies demonstrated that pyrimidine metabolism dysfunction is closely associated with cancer progression. Precision cancer medicine arose from several cancer treatments targeting pyrimidine metabolism [40]. However, there are no previous reports about the relationship between uracil and PA. The development of precision lifestyle interventions requires studies that assess whether PA could alter uracil [40]. Glycerate is a hydroxy monocarboxylic acid anion derived from propionate and is the conjugate base of glyceric acid. A deficiency of human glycerate kinase leads to D-glycerate acidemia/D-glyceric aciduria. Our findings are consistent with a previous study that reported moderate-to-vigorous-intensity leisure-time PA is positively related to glycerate in both AAs and Whites in the respective and pooled samples [41]. Our findings suggest that PA may increase glycerate concentration, and the influence was dependent on regular PA frequency. In addition, glycerate positively correlates with healthy food intake [42] and may confound the relationship between PA and metabolomic abundance. Therefore, a rigorous study design is needed to confirm the effects of PA on metabolite abundances.

The pooled sample analysis revealed that AA but not White participants who exercised ≥3 times/week had a lower abundance of salicyluric glucuronide and a higher abundance of orotate than participants who reported no exercise. Salicyluric glucuronide is a metabolite of salicylic acid and aspirin and does not have a previously reported association with exercise. Orotate, or orotic acid, is classified as a pyrimidine monocarboxylic acid and is an uracil molecule that bears a carboxy substituent at position C-6. Previous studies reported that orotate might improve PA tolerance in trained athletes or patients with coronary artery disease [43,44].

In addition, compared to those who reported no exercise, we found higher abundances of threonate and GPE and a lower concentration of adenine in those who exercised ≥3 times/week in Whites but not in AAs. Adenine combines with the sugar ribose to form adenosine, which can bond with one to three phosphoric acid units, and yield adenosine monophosphate (AMP), adenosine diphosphate (ADP), and adenosine triphosphate (ATP). Our findings are consistent with animal studies. For example, an animal model study found that swimming exercises in rats with adenine-induced chronic kidney disease may result in favorable blood pressure compared to their control counterparts [45], suggesting that exercise may counteract adenine. In addition, our findings could be consistent with those reported in another human study. Zarebska and colleagues [46] tested the effect of exercise on adenine nucleotide levels in 20 endurance-trained adults. Blood plasma was collected at rest, during submaximal and maximal cycle ergometer exercise, and after early recovery. The study reported that plasma ATP and ADP concentrations increased up to 2.5-fold during maximal exercise. Even though not reported, an ATP and ADP increase could potentially result in an adenine decrease, one of the components forming ATP and ADP. Further study is needed to explore PA interventions on adenine concentration and how the changes may relate to health outcomes.

Regarding threonate, the current study findings are consistent with another study reporting that threonate was positively related to PA energy expenditure which was calculated by PA levels objectively measured by ActiGraph in an Asian population [47]. A positive relationship between threonate and a healthy diet may exist. For example, in AAs and Whites, the Healthy Eating Index and Dietary Approaches to Stop Hypertension (DASH) emphasize higher healthy food consumption (e.g., whole grains, fruits, and vegetables) and lower consumption of processed food reportedly have a positive relationship with threonate abundance [48]. Future studies may consider PA and dietary intake when assessing changes in threonate concentration, especially when examining in different populations. GPE is a lipid metabolite of the plasmalogen pathway. Plasmalogens act as an endogenous antioxidant produced by peroxisome, and plasmalogen production is a compensatory mechanism that protects the organism against higher oxidative stress [42]. It is reported that significantly higher GPE was found in athletes who performed high cardiovascular demand (CD; increased stroke volume and blood pressure) sports compared to low-moderate CD athletes, which may potentially place the high CD group at higher risk of cardiovascular disease [49]. The study concluded that the application of metabolites may help avoid potential disorders associated with excessive training. However, the study did not quantify the levels of GPE, and future studies could further assess the harmful threshold of GPE to avoid excessive training or physical activity. Diet studies also assessed GPE. For instance, one study found that the DASH diet is significantly negatively related to GPE compared to either a usual diet (macronutrient intake similar to average US consumption) or a fruit/vegetable diet in adults [50]; consistently, another study reported GPE was positively associated with dietary intake of red and processed meat and CKD progression in children with chronic kidney disease [51]. Further exploration of how multiple lifestyle behaviors, such as PA and diet, alters GPE concentration is warranted.

Notably, we only found significant alterations between those who reported no regular exercise and those who exercised ≥3 times/week. There was a trend of alterations in abundance, but not significant, between those without regular exercise and exercised 1–2 times/week and between those who exercised 1–2 times/week and exercised ≥3 times/week. The findings may suggest that metabolite responses to PA are dose sensitive. These findings are particularly important as they may help identify precision PA interventions when we further understand the pathophysiological mechanism of each metabolite, such as function, normal range, and interaction with other metabolites.

In addition, the abundance of some metabolites changed in African Americans but not in Whites, and vice versa. Some possible reasons might be the population differences in genes [52], and environmental and other lifestyle factors (e.g., diet). For instance, in the current study, we found that BMI was different across PA levels in Whites, but not in AAs; this could be partially attributed to potentially different dietary patterns, which was not assessed in this study. Particularly, racial/ethnic groups not only differ in biological traits, but also in social, economic and cultural backgrounds [38,39]. Historical and contemporary disparities in access to resources [53], living environment [54], discrimination [55], and stress [56] disproportionately affect AA communities in the US. These social economic and cultural factors may contribute to observed differences in metabolomic profiles of AAs and Whites [23]. These factors may also influence lifestyles such as the dietary patterns of the study participants [57,58]. Although not assessed in the current study, these factors (e.g., exposure to discrimination or chronic stress) could potentially confound or modify the association between physical activity and metabolomic profiles. Furthermore, the metabolomic profile reflects the interaction of genes, physical and social environments, and lifestyles [9]; it also reflects one’s health/disease status during the life course journey [59]. Therefore, a big data approach to comprehensively, dynamically, and relatively precisely collect all relevant data is warranted to advance our knowledge and understanding of the human metabolomic profile and its responses to modifiable factors, such as lifestyles, and physical and social environment. Such an approach may provide profound implications for developing precision interventions to decrease health disparities.

## Limitations

The study is one of the first to assess the relationships between PA levels and metabolites in a racially/ethnically diverse population with an untargeted approach. Interpretation of the study findings requires consideration of the following limitations. First, PA was assessed by self-reported regular exercise frequency which might be subject to recall bias. Second, we only assessed regular weekly exercise frequency and did not consider the exercise type and intensity in the current study. However, evidence indicates that weekly exercise frequency is essential for health benefits [60,61]. Third, although we included various covariates in the models, other factors (e.g., dietary intake, sedentary behavior, diseases), which might be related to metabolite alternations were not included [7]. Fourth, given the study’s cross-sectional nature, causality cannot be inferred. Fifth, we only included White and AA men from one geographic area; the generalizability of the findings to women and other populations needs further investigation. Since there might be gender differences in metabolites between women and men, we will assess PA and metabolites in future studies should data be available. Sixth, looking at each racial/ethnical group might have reduced the power to detect some effects in a subgroup. However, the trends of metabolite alterations with exercise levels were in the same direction among all examined groups. Therefore, studies with larger sample sizes and rigorous study designs are needed to investigate whether metabolite responses to PA differ in other populations.

## Conclusion

Compared to those who reported no regular exercise engagement, those who exercised ≥3 times/week had higher uracil, orotate, GPE, threonate, and glycerate metabolite abundances and a low abundance of salicyluric glucuronide and adenine. The findings are primarily consistent with previous reports in other populations. However, in either pooled outcomes or separated outcomes in respect of Whites and AAs, there were no significant differences in the abundances of the seven metabolites between those who reported no exercise and those who exercised 1–2 times/week, as well as between those who exercised 1–2 times/week and exercised ≥3 times/week. These findings suggest that metabolomic responses to exercise might be dose sensitive, which may advance our knowledge of guiding precision PA interventions. In addition, other factors such as dietary intake have been reported to significantly influence some metabolites (e.g., threonate, glycerate, GPE) associated with PA levels [48]; AAs and Whites shared similar alterations in some metabolites but not all. Therefore, studies with rigorous study designs are needed to elucidate the relationship between PA and metabolite responses for precision PA intervention development. Furthermore, a big data approach to comprehensively, dynamically, and relatively precisely collect all relevant data could further advance our knowledge and understanding of the human metabolomic profile and its responses to modifiable factors to reduce health disparities.

## Abbreviations

PA: physical activity
AA: African American
LOS: Louisiana Osteoporosis Study
BMI: body mass index
GPE: 1-(1-enyl-palmitoyl)-2-oleoyl-GPE (P-16:0/18
IRB: Institutional Review Board
QC: qualitative control
MS: mass spectroscopy
HESI-II: heated electrospray ionization
LIMS: laboratory information management system
AMP: adenosine monophosphate
ADP: adenosine diphosphate
ATP: adenosine triphosphate
DASH: dietary approaches to stop hypertension
